# Objectum sexuality: A sexual orientation linked with autism and synaesthesia

**DOI:** 10.1038/s41598-019-56449-0

**Published:** 2019-12-27

**Authors:** Julia Simner, James E. A. Hughes, Noam Sagiv

**Affiliations:** 1 School of Psychology, Pevensey Building, University of Sussex, Brighton, BN1 9QJ UK; 2 Centre for Cognitive Neuroscience, College of Health and Life Sciences, Brunel University, London, Uxbridge, UB8 3PN UK

**Keywords:** Psychology, Human behaviour

## Abstract

Objectum-sexuality (OS) is a sexual orientation which has received little attention in the academic literature. Individuals who identify as OS experience emotional, romantic and/or sexual feelings towards inanimate objects (e.g. a bridge, a statue). We tested 34 OS individuals and 88 controls, and provide the first empirical evidence that OS is linked to two separate neurodevelopmental traits - autism and synaesthesia. We show that OS individuals possess significantly higher rates of diagnosed autism and significantly stronger autistic traits compared to controls, as well as a significantly higher prevalence of synaesthesia, and significant synaesthetic traits inherent in the nature of their attractions. Our results suggest that OS may encapsulate autism and synaesthesia within its phenomenology. Our data speak to debates concerning the biological underpinnings of sexuality, to models of autism and synaesthesia, and to psychological and philosophical models of romantic love.

## Introduction

Categories or continua of sexual orientation are recognised in social, psychological, political and biological sciences. One orientation almost entirely absent from this research arena is *objectum-sexuality* (OS; also known as *objectophilia*). OS individuals describe experiencing emotional, romantic and/or sexual feelings towards inanimate objects or structures. For example, Eija-Riitta Berliner-Mauer has described her romantic attraction towards the Berlin Wall^1^ and others have written similarly about their feelings towards a range of objects (e.g., a bridge, a fence, a statue, an electronic soundboard)^2^. Our background in synaesthesia^3,4^ and autism^5,6^ led us to recognise potential features of both conditions from anecdotal descriptions given by OS individuals–and these features have also been noted by others^7^. For example, one survey^7^ found that a high number of OS individuals (six out of 21) anecdotally reported autism, a set of neurodevelopmental traits that encompass difficulties in social communication, repetitive/routine behaviours, special interests, and sensitivity to sensory stimuli^8^. Although this earlier survey included no significance tests or baselines, it is possible calculate the prevalence of autism within that OS group (28.57%), and this is significantly higher than the general population estimate of 1.46%^9^ (χ^2^(1) = 16.14, *p* < 0.001, 95% CI [0.09, 0.50]). Another neurodevelopmental trait potentially relevant to OS is synaesthesia^10^, which affects around 4% of the general population^11^ but occurs at higher rates in autism^6,12,13^. Synaesthesia gives rise to an unusual fusing of sensations–for example, experiencing a sense of colour when reading letters or numbers (*grapheme-colour synaesthesia*^14^). Important for our purposes here, is that some forms of synaesthesia imbue inanimate objects with genders or personalities. Hence people with *object-personification synaesthesia*^15,16^ might feel that their house-keys are female, or that their pocket watch is shy. Other triggers are linguistic sequences such as letters and numbers (e.g., J might feel motherly, 7 might feel selfish; and this is known as *grapheme-personification synaesthesia* or *ordinal linguistic personification*^4,17^). These synaesthetic personifications have known neurological roots^16,18^ and are reminiscent of descriptions given anecdotally by OS individuals about the objects they are attracted towards. Given the potential overlap between OS, autism, and synaesthesia, our study is the first to explore the relationship among them, using empirical behavioural testing.

In our study we tested 34 self-identified OS individuals and 88 controls, using questionnaires and objective measures related to OS, autism and synaesthesia. If OS is linked with autism, we predict significantly higher rates of diagnosed autism in our OS group, compared to controls and population baselines^9^. We will also use the Autism Spectrum Quotient^19^ (AQ) to measure autistic traits, and predict that the OS group will show significantly higher autistic traits compared to controls. We will also investigate a possible link between OS and synaesthesia, and make several predictions. If OS is linked with synaesthesia, we first predict that OS individuals should describe complex personality traits for their object-partners, similar to those found in object-personification synaesthesia^15,16^. These complex synaesthesia-like percepts should also be *consistent over time*, which is the ‘gold standard’ hallmark of synaesthesia^20,21^ (i.e., genuine synaesthetic associations remain unchanged when repeatedly probed). We therefore followed widely-accepted synaesthesia protocols^3,22^ to determine whether OS individuals show the synaesthetic hallmark of consistency-over-time in the personalities they attribute to objects. To do this, we first asked OS individuals whether they felt personifications in their object-partners (i.e., the objects they were attracted towards). We then assessed the consistency of these personifications by giving a surprise retest 30 minutes later. In our test and retest, OS individuals described their object-partners using 44 personality adjectives (e.g. *outgoing*, *conscientious*, *artistic*) which they rated on a 5-point Likert scale from “Definitely disagree” to “Definitely agree”. This approach maps personality into five dimensions (*Openness to Experience*, *Contentiousness*, *Extraversion*, *Agreeableness*, *Neuroticism*^23^) based on the Big-5 personality model^24^. Control participants were asked to invent personifications for their “most-loved or favourite object” via the everyday mechanisms of anthropomorphism (i.e., the common tendency to attribute humanlike characteristics to nonhuman agents; e.g., “I love my car but she’s temperamental”). In summary, both groups described personalities for objects in a test and retest, and we compared each group’s consistency-over-time (a hallmark of synaesthesia).

If OS is linked with synaesthesia we also predict that OS individuals may show a more general synaesthetic phenotype, and therefore present with additional forms of synaesthesia. Epidemiological studies show that multiple forms of synaesthesia tend to co-occur within the same individual^3^. For example, synaesthetes with grapheme-personification synaesthesia have significantly elevated rates of grapheme-colour synaesthesia^17^. We therefore screened our participants for two additional forms of synaesthesia, using objective diagnostic tests for grapheme-personification synaesthesia^25^ and grapheme-colour synaesthesia^26^. We chose grapheme-colour synaesthesia because it is the most widely tested and well-understood variant of synaesthesia and we chose grapheme-personification synaesthesia because it shows phenomenological similarities with object personification. If OS is related to synaesthesia, we predict higher rates of these synaesthesias within OS individuals.

In summary, if OS is linked with autism, we predict higher rates of autism diagnosis and autism traits in OS individuals, compared to population and control baselines. If OS is linked to synaesthesia, we predict that OS individuals will experience complex personality traits for their admired objects (as found in object-personification synaesthesia), a high level of consistency when describing these personality traits (as found in synaesthesia more broadly), and a higher prevalence of other synaesthesias (grapheme-personification or grapheme-colour synaesthesia) compared to population and control baselines.

## Methods

### Participants

We tested 122 participants comprising 34 OS individuals (18 female, 5 male, 11 other; mean age 32.85, range 17–67, SD = 12.88) and 88 controls without OS (63 female; mean age 19.15, range 18–24, SD = 1.18). OS is extremely rare and our study recruited the largest cohort of this population in the scientific literature to date. Power analyses showed that 20 OS participants and 50 controls would be sufficient to detect differences between two independent proportions (e.g., comparing autism diagnoses across groups) with 80% power, using a previous prevalence of autism in OS of 28.57%^7^. Likewise, 23 OS participants and 59 controls would be sufficient for detecting mean differences between two independent groups (e.g., comparing AQ differences across groups) with 80% power, with a predicted medium-to-large effect size (*d* = 0.7). Here, a medium-to-large effect size is considered reasonable given that we expect OS participants to score highly on tests of autism symptoms, and in consistency for object-personalities (see below).

OS individuals were recruited from online communities of individuals self-identifying as OS (e.g., www.objectum-sexuality.org). Controls were recruited from the University of Sussex community and participated for course credit. The OS group were older than controls (*t*(33.21) = −6.20, *p* < 0.001) but this difference falls in a conservative direction (i.e., the traits we seek in OS *decrease* rather than increase with age^27–30^). Subsets of OS participants (n = 23–34) completed different elements of our study so we preface our methods below with participant numbers for each test. Participants were tested via our online testing portal (www.syntoolkit.org) accessed via a link embedded within our digital recruitment postings. To avoid recruitment bias neither the advert nor information sheet mentioned synaesthesia or autism. Our study was approved by the *Cross-Schools Science and Technology Research Ethics Committee* at the University of Sussex and the study was conducted in accordance with the ethical standards laid down in the 1964 Declaration of Helsinki. All participants provided their full informed consent prior to taking part in the study.

## Materials and Procedure

Participants completed the following tests (in the order described further below):

### Autism screening

[Part 1] Participants (n = 34 OS; n = 88 controls) were asked whether they had received a “*formal diagnosis of a developmental disorder in the past?”* with button options given as “*Autism, Asperger Syndrome, Pervasive Developmental Disorder Not Otherwise Specified, Other, None”*. Participants were also given the opportunity to provide any further details about their autism diagnosis in an optional text-box. [Part 2] Participants also completed the *Autism Spectrum Quotient*^19^ (*AQ*; n = 26 OS; n = 88 controls) which is a 50-item questionnaire measuring autistic traits in adults along five subscales: *Social Skills, Attention Switching, Attention-to-detail, Communication* and *Imagination*. Example items include “*I find it difficult to work out people’s intentions*”, and “*I tend to notice details that others do not*”. Participants respond to each statement on a 4-point Likert scale (*Definitely Agree*, *Slightly Agree*, *Slightly Disagree*, *Definitely Disagree*). Approximately half the questions are reverse coded. Items are scored 1 point if the participant records an autistic trait (e.g., poor Social Skill, good Attention-to-detail) using the ‘slightly’ or ‘definitely’ response. Scores can range from 0 to 50 with a score of 32 or above recognised as a strong indicator of likely autism^19^.

*Sussex Objectum-Sexuality Questionnaire (SOSQ;* n = 34 OS; n = 88 controls) is an in-house measure which first allows participants to self-declare as OS with the question: “*Do you feel romantic and/or sexual attraction towards objects?: Yes, No*”. Participants answering NO to this question are assigned to the control group and are automatically taken to the next stage of study. Participants answering YES are assigned to the OS group and they completed several follow-up OS-related questions (to be reported elsewhere).

*MULTISENSE Test for Object-personification synaesthesia* is an in-house synaesthesia diagnostic based on a previously validated methodology^25^. This diagnostic determines whether individuals experience personifications for non-human objects and whether they show the synaesthetic trait of consistency-over-time (i.e. a high level of consistency in describing the personality of objects over time would suggest their experiences represent object- personification synaesthesia). Part 1 begins for OS participants with the question “*Do you think that your current or most recent object partner/attraction has any personality characteristics?: Yes, No*”. Participants who answer “Yes” complete a personality questionnaire^24^ consisting of 44 randomly ordered personality traits (e.g. *outgoing*, *conscientious*, *artistic*) which OS participants rated to describe their object-partner using a 5-point Likert scale (*“Disagree strongly; Disagree a little; Neither agree nor disagree; Agree a little; Agree strongly”*). Controls were given the same questionnaire but were asked to invent a personality profile for their “*most-loved or favourite object*” (e.g. a favourite chair). In Part 2, all participants repeated the personality questionnaire a second time (and this took place later within the same session; see below) as a surprise retest. The purpose of this surprise retest was to determine the consistency of participants’ responses, given that high consistency is the marker for object-personification synaesthesia^15^. Twenty-three OS participants and 88 controls completed both parts of this test.

*MULTISENSE Test for Grapheme-personification synaesthesia* is a validated diagnostic of grapheme-personification synaesthesia and the full procedure is to be reported elsewhere^25^. As in the test above, this diagnostic determines whether individuals experience personifications (this time for graphemes) and whether they are consistent over time (a hallmark of grapheme-personification synaesthesia). Participants (n = 27 OS; n = 88 controls) began with the opportunity to self-report grapheme-personification synaesthesia from the question “*Do you tend to think of letters or numbers as being like people (i.e. do you associate them with specific personalities or genders, and have always done so before today)?: Yes, No*”. Participants were then asked to indicate whether their pre-existing personifications were for numbers, letters or both (and if both, which was the strongest). Participants then completed a test of consistency in which they saw either numbers 0–9, or letters A-Z, according to their earlier response: i.e., subjects who responded YES to our self-report question were shown whichever set of graphemes (numbers vs. letters) they reported as triggering personalities, or triggering them strongest; controls were randomly allocated to either set. For the purposes of our study all participants completed this test regardless of whether they indicated experiencing grapheme-personification synaesthesia or not.

**Figure 1:**
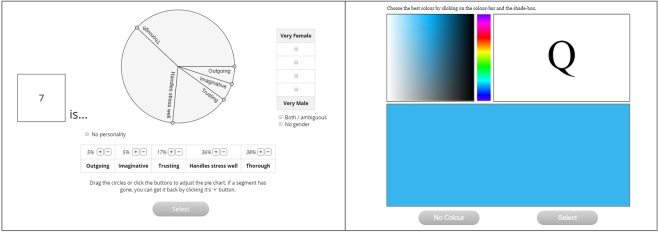
Left panel shows an example trial from our grapheme-personification synaesthesia diagnostic. Participants adjust the segments of the pie-chart to select the personality of the presented grapheme. Participants also used the Likert scale to describe a gender for each grapheme. Right panel shows an example trial from the grapheme-colour synaesthesia diagnostic. Participants adjust the colour-picker to select the associated colour for each presented grapheme. In both cases (left and right panels) graphemes are presented twice (in a test and retest) and we calculate the synaesthetic measure of consistency-over-time. High consistency is the hallmark of synaesthesia.

During the consistency test, graphemes were shown one-by-one in a random order, and participants were required to rate the personality of each grapheme using an adjustable pie-chart of five personality descriptions (*imaginative*, *thorough*, *outgoing*, *trusting*, *handles stress well*; see Fig. 1, left panel) one for each factor of the Big-5 personality model^24^. Participants rated the relative contribution of each trait to the overall personality profile of the grapheme (e.g., This grapheme is 40% imaginative, 20% thorough, 5% outgoing, 10% trusting, 25% handles stress well). Participants also rated the gender of each grapheme from “very male” to “very female” on a 5 point Likert scale. Participants could also select *no personality* and/or *no gender* if they did not experience a personality or gender for a particular grapheme, although participants were instructed to avoid pressing these buttons as much as possible (because we wanted a consistency score for both OS individuals and controls). Each grapheme was displayed twice in a block design resulting in 20 trials for numbers (i.e., 2 × 10), or 52 trials for letters (i.e., 2 × 26). The test generated two output measures, indicating how consistently participants described the personality or gender of their graphemes. Following validated procedures^25^, we calculated consistency (for personality and gender scores separately) by regressing participants’ responses for their first set of personality and gender descriptions against their second set of descriptions. This indicated the degree to which the first set of descriptions were a predictor of the second set of descriptions (as a measure of consistency). Synaesthetes were diagnosed as those scoring at or above the validated threshold for synaesthesia, which was again computed separately for personality ratings and gender ratings. A diagnostic threshold for synaesthesia of ≥34.97 was used for personality scores, while a diagnostic threshold of ≥82.69 was used for gender scores^31^.

*MULTISENSE Test for Grapheme-Colour synaesthesia* is an in-house version of the widely-validated^3,22,32^ diagnostic for grapheme-colour synaesthesia. Its procedure is similar to the synaesthesia diagnostics above^25^ except that it elicits colours for graphemes (rather than personifications). Participants (n = 26 OS; n = 88 controls) first had the opportunity to self-report grapheme-colour synaesthesia given the question “*Do numbers or letters have colour associations (i.e. which you’ve been aware of before now)?; Yes, No*”. Participants responding NO are classified as non-synaesthetes and are not tested further (following standard testing protocols^3,22^). Participants responding YES specify whether they experience colours for numbers, letters or both, and in the subsequent consistency test they are shown whichever graphemes they reported. In consistency testing, participants pair each grapheme with a colour, using an interactive colour-picker (see Fig. 1, right panel). Graphemes are each shown twice in a block-randomised design, and test-scores are an indication of the distance in colour space between each presentation of the same grapheme (e.g., the colour-distance between the two presentations of A). Smaller scores represent smaller distances, hence greater consistency-over-time. Scores are averaged across all trials and standardised; following validated methods^3,33^, synaesthetes were diagnosed as those scoring within the recognised synaesthetic window (i.e., ≤1.43^34^, where low scores indicate the high consistency typical of synaesthesia).

### Order of experimental tests

Tests were presented in the following order: Autism screening [Part 1], SOSQ, Object-personification [Part 1], grapheme-personification synaesthesia test, grapheme-colour synaesthesia test, Autism screening [Part 2], Object-personification [Part 2].

## Results

### Links between objectum sexuality and autistic traits

**Figure 2:**
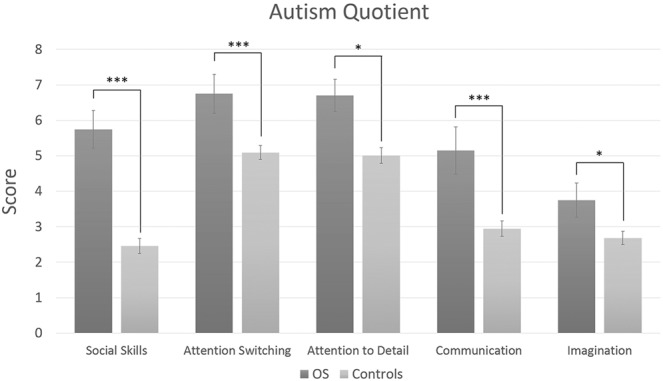
Mean AQ scores for OS and control participants. Higher scores represent more autistic-like traits (i.e., poor social skills, attention switching, communication, imagination; good attention to detail). Error bars show standard errors of the means (SEMs). Asterisks indicate corrected significance at *p < 0.05; **p < 0.01; ***p < 0.001.

In this section, we ask whether OS individuals show higher autistic traits compared to controls. All analyses here and throughout are two-tailed. In our first analysis, a 2 × 5 ANOVA contrasting group (OS vs. controls) and AQ factor (Social Skills, Attention switching, Attention-to-detail, Communication, Imagination) showed a main effect of group in that OS individuals possessed overall stronger autistic traits than controls (F(1,112) = 50.33, *p* < 0.001, ηp2 = 0.31; see Fig. 2). In addition we found a main effect of AQ factor (F(4,448) = 35.55, *p* < 0.001, ηp2 = 0.24) as well as an interaction between group and factor (F(4,448) = 6.96, *p* < 0.001, ηp2 = 0.06). Exploring this interaction further showed that OS participants scored higher than controls in every AQ factor (all corrected *ps* < 0.05) but particularly so in the social skills factor as indicated by its ‘very large’ effect size (*d* = 1.55^34,35^ all other effect sizes were ‘medium’ (*d* ≥ 0.5) to ‘large’ (*d* ≥ 0.8); see *Supplementary Information* Table S1).

### The prevalence of autism in objectum sexuality

In addition to heightened AQ scores, 13 out of 34 OS participants (38.24%) but none of our 88 controls reported having an official diagnosis of autism, and this difference was significant (χ^2^(1) = 33.75, *p* < 0.001, 95% CI [0.22, 0.56]). OS individuals also had significantly more diagnoses of autism compared to published epidemiological data from the general population^9^ (1.47%; χ^2^(1) = 30.60, *p* < 0.001, 95% CI [0.20, 0.55]). Note that all chi-square analyses here and throughout the manuscript are performed with the more conservative Yates continuity correction where expected cell counts fall below five observations.

Given our online methodology, we were not able to perform independent clinical autism assessments. We therefore ran additional analyses showing that even when we use more conservative methods for classifying participants as autistic, our results remain the same. Our first additional analysis classified participants as autistic only if they satisfy two criteria: 1) they self-reported a formal diagnosis of autism (as above) AND 2) they gave further spontaneous information about their diagnosis via our optional text-box (e.g., their age at diagnosis). Following this new autism classification, we continued to find significantly higher rates of autism in our OS group (8 out of 34; i.e., 23.53%) compared to our control group (0 out of 88; χ^2^(1) = 18.49, p < 0.001, 95% CI [0.10, 0.42]). This more conservative rate of autism in OS was again significantly higher than published epidemiological data from the general population^9^ (1.47%; χ^2^(1) = 14.39, p < 0.001, 95% CI [0.08, 0.40]). Finally, we also analysed our AQ data and classified participants as autistic only if they scored above the recognised indicative threshold for autism (i.e. ≥ 32 points). Once again we found a significant group difference: there were 10 out of 34 OS participants classified as autistic (29.41%), compared to 2 out of 88 controls (2.27%; χ^2^(1) = 17.42, p < 0.001, 95% CI [0.12, 0.46]). Moreover, rates of autism in the OS group were once again significantly higher than published epidemiological data from the general population (1.47%; χ^2^(1) = 20.60, p = < 0.001, 95% CI [0.13, 0.46]). In summary, we find higher rates of autism/ autistic traits for OS individuals in all analyses.

### Links between objectum sexuality and synaesthesia

We first asked whether OS participants experience synaesthesia-like personalities from objects. We therefore analysed scores from the Multisense Test for Object-personification synaesthesia. During this test, OS participants had rated the personality of their object-partner while controls rated the personality of their ‘most-loved or favourite object’. Both groups were then given a surprise retest. We calculated the consistency of object-personalities by regressing participants’ responses for their first set of personality descriptions against their second (retest) set of descriptions. OS participants were significantly more consistent over time (*t*(109) = 3.63, *p* < 0.001, *d* = 0.92, 95% CI [9.13, 31.11]) in their object-personality descriptions (M = 69.5%, SD = 18.5%) compared to controls (M = 49.4%, SD = 24.8%) supporting the hypothesis that OS feelings might stem from what can be recognised as object-personification synaesthesia.

Next, we investigated whether OS was related to a more generalized synaesthetic phenotype, by looking at whether OS individuals also have *other* variants of synaesthesia. Here we diagnosed grapheme-personification synaesthesia and grapheme-colour synaesthesia, using recognised protocols and previously validated thresholds^22,31,33^. We found higher rates of both types of synaesthesia in our OS group.

Considering first grapheme-personification synaesthesia, we found higher rates of synaesthesia in the OS group compared to controls (although only in gender scores, see Table 1; we remind the reader that it is possible to diagnose grapheme-personification synaesthesia either for grapheme genders (e.g., letter A is female) or for grapheme personalities (e.g., letter A is shy)).

**Table 1:** Rates of grapheme-personification synaesthesia in OS and Control groups.

Type of personification	Prevalence of grapheme-personification synaesthesia		Statistical test
	OS group	Control group	
Genders	29.63%	10.23%	χ^2^(1) = 6.17, *p* = 0.013, 95% CI [0.03, 0.41]
Personalities	2.9%	1.1%	χ^2^(1) = 0.00, p = 1.00

Table shows that the OS group had a higher prevalence of grapheme-personification synaesthesia (for grapheme-genders but not grapheme-personalities). See *Supplementary Information - Prevalence of grapheme-personification synaesthesia* for further details regarding these prevalence estimates.

Since seven of our OS participants left our study before taking this (grapheme-personification) synaesthesia test, we repeated our analysis with the conservative assumption that all were non-synaesthetes and this still resulted in a strong trend for a higher prevalence of confirmed grapheme-personification synaesthesia for gender associations in the OS group compared to controls, χ^2^(1) = 3.618, p = 0.057, 95% CI [−0.004, 0.30].

In addition to the above finding, we also found significantly elevated rates of grapheme-colour synaesthesia, which was 14 times higher in OS individuals (15.4% i.e., 4 out of 26) compared to controls (1.1% i.e., 1 out of 88 controls; χ^2^(1) = 6.62, *p* = 0.01, 95% CI [0.02, 0.35]) and 11 times higher than the known population-wide baseline (of 1.4%^3^; χ^2^(1) = 25.34, p < 0.001, 95% CI [0.04, 0.34]). Since eight of our OS participants left our study before taking the grapheme-colour synaesthesia test, we repeated our analysis with the conservative assumption that all were non-synaesthetes and our findings remained significant (χ^2^(1) = 4.60, *p* = 0.032, 95% CI [0.01, 0.27]). Finally, we also considered whether OS individuals might have synaesthesia simply because they may also have autism, given that synaesthesia and autism are co-morbid^6,12,13^. But rates of grapheme-colour synaesthesia were still significantly higher than controls even when considering only OS individuals who did *not* have autism (14.29%; 3 out of 21; χ^2^(1) = 4.99, p = 0.025, 95% CI [0.01, 0.36]).

Further above we have seen that OS individuals give personality traits for their object-partners that are consistent over time. We point out that the high consistency in reports of personalities described by OS individuals for their object-partners were not simply some heightened ability to remember personality traits: OS individuals were no more consistent than controls when attributing personalities to letters or numbers (revealed by a lack of group differences in prevalence for personality ratings during the grapheme-personification test). This suggests that OS individuals are not better memorisers of personalities in general, but tend to have genuine object-personification synaesthesia, as well as elevated rates of both grapheme-personification synaesthesia (for genders only) and grapheme-colour synaesthesia.

## Discussion

To date, only one other study^7^ has addressed objectum sexuality (OS) and our research is the first to take an empirical behavioural approach in studying this form of sexual orientation. We recruited a group of individuals who self-identify as OS and a group of non-OS controls. Our study was motivated by the hypothesis that autism and synaesthesia (i.e., object-personification synaesthesia) might play a role in OS, at least for some OS individuals.

We found a number of significant relationships between OS and synaesthesia. Firstly, we found that the romantic affections of OS individuals towards objects do appear to be driven, at least in part, by object-personification synaesthesia. This was revealed by the fact that OS individuals do tend to sense personality traits in their object-partners, and that these show the synaesthetic hallmark of consistency-over-time (i.e. the OS group were significantly more consistent compared to controls when assigning personalities to inanimate objects). We propose that synaesthetic personalities and genders might increase the anthropomorphic qualities of inanimate objects which could facilitate the development of intimate and romantic feelings over time. Finally, OS was also associated with a broader synaesthetic phenotype in showing significantly elevated rates of two other types of synaesthesia, grapheme-personification synaesthesia and grapheme-colour synaesthesia.

We found too that rates of diagnosed autism were over 30 times higher in OS individuals than otherwise expected (or 14 times higher even with our most conservative estimate). Differences were most associated with the Social Skills sub-scale of the AQ (finding social situations difficult or unenjoyable, with a preference for objects over humans in one AQ question). Poor inter-human relationships may be relevant to why OS individuals develop relationships with objects although it is unclear whether poorer social skills might be a contributing factor towards OS or indeed a consequence. For instance, objectum sexuals experience considerable social stigma attached to their orientation^2^ which could affect social skills through an avoidance of interactions with other people. Relatedly, anthropomorphizing has been suggested to be a compensatory mechanism when individuals experience increased levels of loneliness^36^ and autistic individuals report higher ratings of loneliness compared to controls^37^. Other autistic traits might also facilitate feelings of intimacy with objects: a strong need for routine might be fulfilled more easily in relationships with objects rather than people, while higher attention-to-detail and a tendency for special interests could promote heightened focus on, or appreciation for particular objects^7^. Finally, synaesthetic personifications may further reinforce this attention since objects with positive synaesthetic personalities are even known to bias eye-movements for synaesthetes^15^. Nonetheless, we point out that just over one third of OS individuals did *not* report a diagnosis of autism and did not score above the AQ threshold (although a number of these scored closely). It is therefore possible that autism is not a necessary prerequisite for OS in all cases. Nevertheless, our current data suggests that OS, autism, and synaesthesia are linked at multiple levels and future research could focus on delineating the precise nature of how autism and synaesthesia interact in OS.

An important methodological consideration is the fact that our OS participants were recruited from forums online. We took this approach because it would have been difficult to achieve in-person testing given that OS is rare, internationally distributed, and without any regular real-world meeting place (as far as we are aware). More importantly, our participants were reassured by the protection of anonymity, meaning that in-person testing would inevitably have reduced sample size. Importantly, one might wish to argue that recruiting from online platforms could disproportionally attract people with social difficulties (and thereby elevate AQ scores). However, we point out that the OS group scored higher than controls across *all* factors of the AQ (Social skills, Attention-switching, Attention-to-detail, Communication, and Imagination) suggesting that our results are not skewed by the social traits of people drawn to online forums. Relatedly, one might wish to argue that rates of autism may be a priori higher on such forums, but we point out that rates of diagnosed autism in OS individuals was almost 40%, a prevalence that is hard to explain away as the result of our online recruitment methods.

In addition to the above consideration, we note that our online methodology did not allow for us to perform clinical diagnoses of autism; we therefore took participants’ reports of autism at face-value. Nevertheless, we were careful to ask about “*formal”* diagnoses, and we replicated our findings on a sub-set of participants whose formal diagnoses were backed-up with substantive details (i.e., when their diagnoses had taken place). We further replicated using AQ scores (based on the autism threshold of ≥32^19^). Given these conservative approaches, we are confident our classifications of autism are accurate, and we also point out that our findings are replicated by our analysis of autism rates in the cohort tested by Marsh^7^ (see Introduction). Finally, we address one last concern, that AQ scores in OS participants could be circular, given that one question in the AQ (out of 50) could potentially define OS (i.e., the reverse-coded “I find myself drawn more strongly to people than to things”). If we remove this question from our AQ analysis we still observe the same pattern of results (i.e. OS individuals still score significantly higher on the Social skills factor of the AQ with a *large* effect size; and 10 OS individuals compared to 2 controls still score above the diagnostic threshold ≥32 on the AQ overall; see *Supplementary Information* Table S2). Nonetheless, even aside from the ‘literal’ question described above, it could be argued that high scores on the social factor of the AQ might simply reflect the social challenges of identifying as OS. Importantly however, OS individuals were elevated across *all* sub-scales of the AQ. We are therefore confident that our results speak to rates of autism, rather than being a circular finding about OS per se.

Our data speak to biological^38^, psychological^39,40^ and philosophical^41^ models of sexuality or romantic love. We inform biological theories by showing that certain sexual orientations can be found at higher rates within recognised neurodevelopmental conditions with known genetic traits^10,42^ and specific neurological profiles^43,44^ independent from the sexual orientation itself. Fifty-three percent of our OS cohort was characterised as having either synaesthesia and/or autism (from diagnosis or elevated AQ scores, with a further 9% scoring close to threshold), placing these well-understood neurodevelopmental conditions firmly at an intersection with this sexual orientation. Our data also support one very recent suggestion^45^ that autism might dictate sexual preference to some extent, and suggest the need for raised awareness among medical practitioners to support and promote inclusion for autistic people regarding orientation. Our data also speak to models of synaesthesia showing synergistic outcomes when synaesthesia and autism co-occur within individuals^6^ and they speak to the study of anthropomorphism^36^ by validating the use of detailed personality questionnaires^46^ for non-human targets. We suggest that this methodology might be further exploited to ask whether personality profiles for objects in OS (or indeed in general anthropomorphism) are random or ordered. For example, our methods could test whether object-personalities reflect the personality traits of those humans making judgements – as would be predicted if object personifications arise from mechanisms within self-other judgements^16,47^. Finally, our data address psychological^39^ and philosophical^41^ models of romantic love which have traditionally been defined around personhood (i.e., a feeling of pleasure derived from an attraction towards another *person*). Our findings suggest, crucially, that *personification* rather than *personhood* may be the necessary pre-requisite for romantic love to arise.

Our study is the first behavioural treatment of OS in the scientific literature. Although inquiry into OS has gained little traction in modern science, wider understanding would be particularly beneficial given that objectum sexuals are often marginalised for their orientation^2^. In summary, we have shown links between OS, synaesthesia and autism, and propose that increased anthropomorphizing in OS may sometimes occur as a result of differences in social cognition and other autism-driven traits as well as object-personification synaesthesia.

## Supplementary information

Supplementary Information

## Data Availability

The data supporting this study are available via the UK Data Service.
