# Supplementary material for: Objectum sexuality: A sexual orientation linked with autism and synaesthesia
Source: Sci Rep. 2019 Dec 27;9:19874. doi: 10.1038/s41598-019-56449-0 (PMC6934473; doi:10.1038/s41598-019-56449-0)
Supplement: Supplementary file 1 — Supplementary Information [file 41598_2019_56449_MOESM1_ESM.pdf]

Objectum sexuality: A sexual orientation linked with autism and synaesthesia

Julia Simner[1], James E.A. Hughes[1] & Noam Sagiv[2]

[1]School of Psychology, Pevensey Building, University of Sussex, Brighton. BN1 9QJ. UK.
[2]Centre for Cognitive Neuroscience, College of Health and Life Sciences, Brunel University, London, Uxbridge. UB8 3PN. UK.

Supplementary Information

MULTISENSE Test for Object-personality synaesthesia

The test asks participants to describe the personality of their current or most-recent object partner/attraction. This section is presented twice to participants during the study, with an interval of approximately 30 minutes between the two presentations. The purpose of presenting this test twice was to allow us to calculate consistency scores between the test and retest (e.g., how consistent was the participant's personality for the letter A?). We could then compare this consistency across OS and control groups. To elicit these personalities, we used an adapted version of the Big Five Inventory (BFI), which is a measure typically used to measure personality traits in people[1]. This adaptation required participants to rate not people (as the questionnaire had originally been designed) but objects, and was also marginally abridged in wording for conciseness (e.g., "Is reserved" → "reserved").

Test description and items as they appeared in the study

Below we present the full instructions and items used in our test, as seen by participants:

The following questions are all concerned with your 'current object partner', by this we mean the object that you are currently in a romantic and/or sexual relationship with (or if you are not currently in a relationship then your most recent object-relationship). If you have never been in an object-relationship then please think of the object you are currently attracted to (please pick only one object).

We would now like you to describe the personality of the object that you described above (i.e. your current or most recent object-partner/attraction). Please consider the following personality traits below, and decide whether they apply to your object by selecting one of the five options.

My current/most-recent object partner/attraction is...

	Disagree strongly	Disagree a little	Neither agree nor disagree	Agree a little	Agree strongly
Thorough	◉	○	○	○	○
Lazy	◉	○	○	○	○
Reserved	○	○	○	○	○
Outgoing	○	○	○	○	○
Handles stress well	○	○	○	○	○
Imaginative	○	○	○	○	○
Critical of others	○	○	○	○	○
Trusting	○	○	○	○	○
Unartistic	○	○	○	○	○
Nervous	○	○	○	○	○
Talkative	○	○	○	○	○
Depressed	○	○	○	○	○
Original, has ideas	○	○	○	○	○
Helpful	○	○	○	○	○
Careless	○	○	○	○	○
Curious	○	○	○	○	○
Energetic	○	○	○	○	○
Starts quarrels	○	○	○	○	○
Reliable	○	○	○	○	○
Tense	○	○	○	○	○
A deep thinker	○	○	○	○	○
Enthusiastic	○	○	○	○	○
Forgiving	○	○	○	○	○
Disorganized	○	○	○	○	○
Worries a lot	○	○	○	○	○
Quiet	○	○	○	○	○
Emotionally stable	○	○	○	○	○
Inventive	○	○	○	○	○
Assertive	○	○	○	○	○
Cold and aloof	○	○	○	○	○
Perseveres	○	○	○	○	○
Moody	○	○	○	○	○
Artistic	○	○	○	○	○
Shy	○	○	○	○	○
Considerate	○	○	○	○	○
Efficient	○	○	○	○	○
Calm	○	○	○	○	○
Prefers routine	○	○	○	○	○
Rude	○	○	○	○	○
Follows through with plans	○	○	○	○	○
Likes to reflect	○	○	○	○	○
Cooperative	○	○	○	○	○
Distractible	○	○	○	○	○
Sophisticated	○	○	○	○	○

Table S1 Statistical outputs from our evaluation of AQ (*Autism Spectrum Quotient*[2]) scores. Table shows p values (Bonferroni corrected), effect sizes, and 95% CI's for each AQ factor (column 1), comparing the OS group to controls (OS show significantly *more* autistic traits across all factors).

AQ factor	*p* value	Cohen's *d*	95% CI
Social skills	< .001	1.552886	2.63758 - 4.53026
Attention switching	< .001	.905811	1.01190 - 2.72936
Attention to detail	= .014	.691021	.49867 - 2.32476
Communication	< .001	1.081296	1.48589 - 4.01236
Imagination	= .045	.560462	.27737 - 1.89745

Table S2 Statistical outputs from our evaluation of AQ scores. Table shows p value (Bonferroni corrected), effect size, and 95% CI for the Social skills factor of the AQ (comparing OS to controls) after the removal of one item within this factor*.

AQ factor	*p* value	Cohen's *d*	95% CI
Social skills	< .001	1.241668	1.85971 - 3.68049

*We removed one question from the Social skills factor that could potentially define OS rather than autism per se: "I find myself drawn more strongly to people than to things" (a reverse-coded item)". The data contained in this table shows reanalysed AQ scores for this factor, after the removal of this one item (all other factors were unaffected). The pattern of results remained the same: i.e., as before, OS show significantly more autistic traits even after removing this item.

Prevalence of grapheme-personification synaesthesia

We point out that the 10.23% prevalence of grapheme-personification synaesthesia (synaesthetic genders for letters/ numbers) in our control population is relatively high, and this is likely due to expected limitations in the test. This test was validated using data from 23 known grapheme-personification synaesthetes and 212 non-synaesthete controls, who were tested by Hughes et al.[3] A ROC analysis (receiver operating characteristics) provided the thresholds reported here (e.g., ≥ 82.69 in gender scores) and these thresholds maximised sensitivity and specificity (for full details, see Hughes et al.). Importantly, the sensitivity of this test was .87 (i.e., it correctly diagnoses 87% of synaesthetes) and its specificity was .83, meaning that it correctly rejects 83% of non-synaesthetes -- but inevitably misdiagnoses a small number. This may be contributing to the prevalence of synaesthesia in our current samples, even controls. Importantly however, the ROC validation confirms that this test is an "excellent" diagnostic of synaesthesia[3], albeit with these limitations -- none of which influence our key findings here (i.e., higher rates of synaesthesia in OS individuals).

References

1. John, O. P. & Srivastava, S. The Big-Five Trait Taxonomy: History, Measurement, and Theoretical Perspectives. in *Handbook of personality: Theory and research* (eds. Pervin, L. A. & John, O. P.) 102–138 (Guilford Press, 1999).
2. Baron-Cohen, S., Wheelwright, S., Skinner, R., Martin, J. & Clubley, E. *The Autism-Spectrum Quotient (AQ): Evidence from Asperger Syndrome/High-Functioning Autism, Males and Females, Scientists and Mathematicians*. *Journal of Autism and Developmental Disorders* **31**, (2001).
3. Hughes, J. E. A., Ipser, A. & Simner, J. The MULTISENSE Test for Ordinal Linguistic Personification. (2018).
